# Pulmonary MRI contrast using Surface Quadrupolar Relaxation (SQUARE) of hyperpolarized ^83^Kr^☆^

**DOI:** 10.1016/j.mri.2013.08.007

**Published:** 2014-01

**Authors:** Joseph S. Six, Theodore Hughes-Riley, David M.L. Lilburn, Alan C. Dorkes, Karl F. Stupic, Dominick E. Shaw, Peter G. Morris, Ian P. Hall, Galina E. Pavlovskaya, Thomas Meersmann

**Affiliations:** a Sir Peter Mansfield Magnetic Resonance Centre, University of Nottingham, Nottingham NG7 2RD, UK; b School of Medicine, University of Nottingham, Nottingham NG7 2UH, UK; c School of Physics and Astronomy, University of Nottingham, Nottingham NG7 2RD, UK; d Nottingham Respiratory Research Unit, University of Nottingham, Nottingham NG5 1PB, UK

**Keywords:** ^83^Kr, Krypton-83, Kr-83 hyperpolarization, Hyperpolarized, Noble gas MRI, Spin polarization, Cryogenic separation, Spin-exchange optical pumping, Nuclear electric quadrupole moment, Quadrupolar relaxation, Surface sensitive contrast, Pre-clinical MRI, Pulmonary MRI, Lung surfactant

## Abstract

Hyperpolarized ^83^Kr has previously been demonstrated to enable MRI contrast that is sensitive to the chemical composition of the surface in a porous model system. Methodological advances have lead to a substantial increase in the ^83^Kr hyperpolarization and the resulting signal intensity. Using the improved methodology for spin exchange optical pumping of isotopically enriched ^83^Kr, internal anatomical details of ex vivo rodent lung were resolved with hyperpolarized ^83^Kr MRI after krypton inhalation. Different ^83^Kr relaxation times were found between the main bronchi and the parenchymal regions in ex vivo rat lungs. The T_1_ weighted hyperpolarized ^83^Kr MRI provided a first demonstration of surface quadrupolar relaxation (SQUARE) pulmonary MRI contrast.

## 1 Introduction

Pulmonary MRI with hyperpolarized (hp) ^129^Xe [1] and hp ^3^He [2] are emerging techniques for spatially resolved measurement of lung function that cannot be obtained by alternative non-invasive methods. Both non-radioactive isotopes have a nuclear spin I = 1/2 that can be hyperpolarized through laser-based methods [3,4] to obtain sufficient MRI signal intensity for high resolution imaging of the lung. Various MRI protocols can be used to generate complementary contrast from the two isotopes. For example, because of its high diffusivity, ^3^He is thus far preferred for contrast relating to changes in alveolar lung structure (i.e. ADC contrast) [5–8]. The ^3^He spin relaxation is more affected by the presence of paramagnetic O_2_ in the gas phase than that of any other noble gas isotope and the ^3^He T_1_ relaxation can therefore be used for partial pressure measurement of pulmonary oxygen [9–11]. On the other hand, the large chemical shift range of ^129^Xe leads to distinguishable MR signals between tissue dissolved and gas phase xenon [12] thus enabling the visualization of gas transport through the parenchyma [13]. The isotope ^129^Xe generally possesses a relatively high solubility, has a relaxation times of T_1_ = 13 s in oxygenated blood [14], and can be functionalized to serve as a biosensor for certain target molecules [15] with potential applications for pulmonary MRI and beyond. The development of hp pulmonary MRI is therefore not only a quest for higher signal intensity and better spatial resolution but also a pursuit for novel sources of contrast that probe different structural and functional aspects of lungs in health and disease [11,16].

Using a third noble gas isotope, namely ^83^Kr, longitudinal (T_1_) relaxation weighted MRI contrast was previously shown to be indicative of the specific surface treatment in a porous model system [17]. Unlike ^3^He and ^129^Xe, the ^83^Kr nucleus possesses a nuclear spin I = 9/2 and thus a non-vanishing electric quadrupole moment that serves as a probe for electric field gradients (EFGs). The EFGs are predominantly generated during brief collision and adsorption events of the noble gas atoms with the surrounding surfaces, resulting in rapid T_1_ relaxation that is detected in the gas phase. The ^83^Kr surface quadrupolar relaxation (SQUARE) MRI contrast is affected by the surface to volume ratio (S/V), surface composition, surface temperature, and surface adsorption of molecules [16–18]. On the downside, quadrupolar relaxation also restricts the hp ^83^Kr signal intensity and applications of hp ^83^Kr MRI were limited thus far to conceptual studies showing low resolution images [17,19] with little chance to provide data about internal structure or function of the lung.

In recent work, spin exchange optical pumping (SEOP) of a mixture of 5% krypton with 95% N_2_ achieved a ^83^Kr spin polarization of P = 26%, corresponding to a 59,000 fold signal increase compared to the thermal equilibrium ^83^Kr signal at 9.4 T field strength [20]. SEOP at low krypton concentration was used because high krypton density [Kr] adversely affects SEOP but, unfortunately, fast quadrupolar driven ^83^Kr T_1_ relaxation in the condensed state generally prevents the cryogenic separation of hp krypton from the gas mixture [21]. The high gas dilution caused a 20 fold reduction of the MRI signal and it is instructional to define the apparent polarization P_app_ that takes the dilution into account [20]:

[1] $$P{}_{\mathrm{app}}=P\cdot \left[\mathrm{NG}\right]/\sum_{i}\left[M_{i}\right]$$

where [NG] is the noble gas density (here, krypton) and [M_i_] refers to the density of other components in the hp gas mixture (i.e. N_2_ in this work). The apparent polarization provides a measure of the expected signal from a diluted hp noble gas. The example above (*P* = 26%) leads to *P*_app_ = 1.3% and thus to the same signal of pure krypton gas with *P* = 1.3% (assuming identical isotopic composition).

As an alternative to dilution, the density [Kr] can be lowered in concentrated krypton mixtures by reducing the SEOP gas pressure [20]. In the current work, this method was modified to extract below ambient pressure hp gas mixture from the SEOP cell followed by compression to ambient pressure for pulmonary imaging. Hp ^83^Kr produced with this method was utilized to study SQUARE contrast in an excised rat lung.

## 2 Materials and methods

### 2.1 ^83^Kr spin exchange optical pumping.

Spin exchange optical pumping (SEOP) with rubidium produced hp ^83^Kr via batch mode as described in detail elsewhere [20]. Spin polarization measurements used natural abundance krypton gas (99.995% purity; 11.5% ^83^Kr; Airgas, Rednor, PA, USA), whereas the MR images presented in this publication utilized enriched ^83^Kr (99.925% ^83^Kr, CHEMGAS, Boulogne, France) for improved signal intensity. A 25% krypton–75% N_2_ (99.999% purity, Air Liquide, Coleshill, UK) mixture was used for SEOP because it was previously proven to lead to high hp ^83^Kr signal intensities [20] and allowed for economical usage of the expensive isotopically enriched ^83^Kr gas.

Spin polarization was determined by comparison of the hp gas signal in a single pulse experiment with that from a thermally polarized krypton gas [20]. In baseline polarization measurements the hp gas was transferred by gas expansion directly into a pre-evacuated borosilicate glass cell located in the r.f. detection coil without usage of the extraction unit. Spin polarization measurements were acquired after 8 minutes of SEOP and images were acquired after 12 minutes of SEOP, corresponding to ~ 80% and ~ 92% of the steady state polarization (reached after 18 minutes [20]) respectively, to reduce experimental time.

### 2.2 HP gas extraction, compression and transfer.

**Fig. 1:**
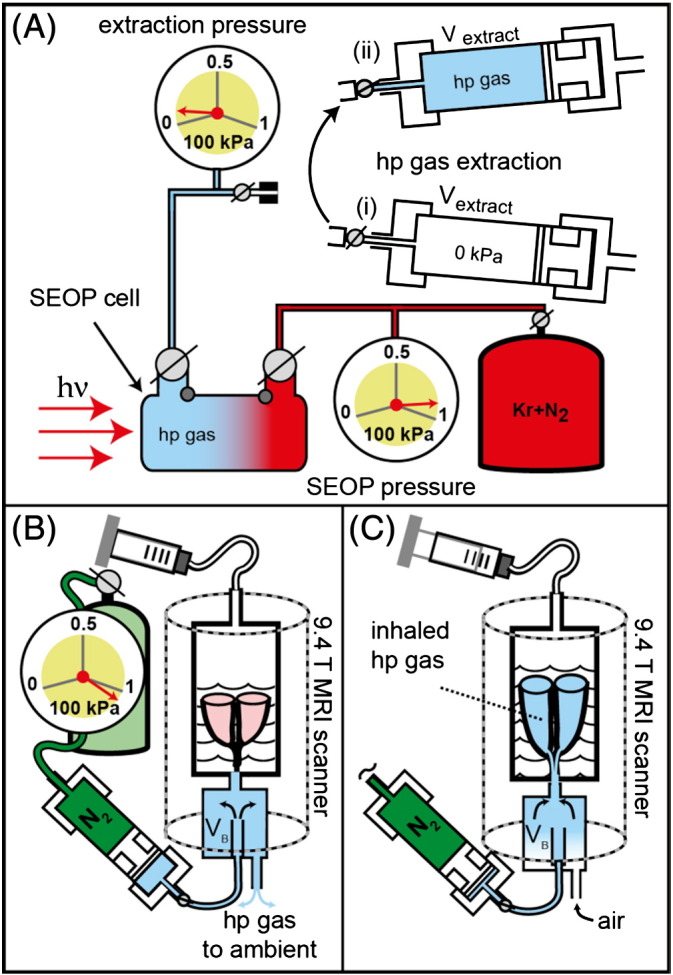
Hp krypton extraction and transfer from the SEOP cell, operating at 90–100 kPa, to the lungs at ambient pressure. (A) A pre-evacuated volume V_extract_ = 790 cm^3^ in the extraction unit (i) was filled to approximately 6 kPa during hp gas extraction (ii). (B) The extraction unit was moved to the MRI scanner and the N_2_ gas operated piston pressurizes the hp gas mixtures to a pressure slightly above ambient. The hp gas was then pushed through connecting tubing into a storage container (V_B_). The lung was located upside down in glucose solution within the breathing apparatus with the trachea connected to V_B_. (C) A slight suction on the breathing apparatus (substituting for the pleural cavity) caused the lung to expand and to inhale the hp gas.

**Fig. 2:**
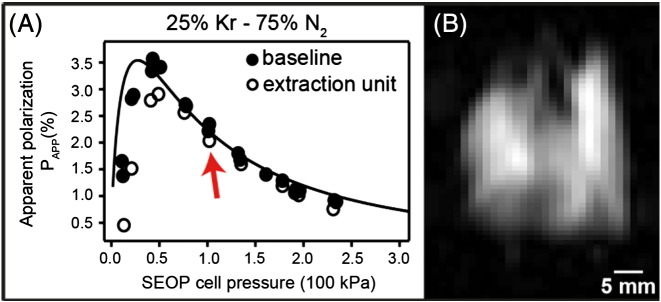
(A) The apparent ^83^Kr spin polarization *P*_app_ as a function of SEOP cell pressure using the extraction unit for compression (open circles) and baseline data without the extraction unit (filled circles). The arrow indicates the pressure used for imaging experiments. Curve fitting was adapted from ref. [20]. (B) Variable flip angle (VFA) FLASH hp ^83^Kr MRI of an excised rat lungs at 9.4 T without signal averaging (NEX = 1, no slice selection, SNR = 51) using isotopically enriched ^83^Kr (99.925%).

To utilize the enhanced ^83^Kr spin polarization of below ambient pressure SEOP [20] an extraction unit was designed and built that extracted the hp gas from the SEOP cell and then delivered the gas for pulmonary imaging as shown in Fig. 1. At 90–100 kPa SEOP cell pressure this method produced approximately 35–40 cm^3^ of hp gas mixture every 12 minutes for lung imaging. Alternatively, in the spin polarization measurements the hp gas was injected into an NMR detection cell to measure the ^83^Kr spin polarization after the compression process (Fig. 2).

### 2.3 HP gas inhalation.

A ventilation chamber with the lung suspended in a 5% glucose solution (weight/volume) (Baxter Healthcare Ltd, Thetford, UK) was placed inside the MR magnet and kept at a constant temperature of 295 K. Active inflation of the lung was achieved by producing a negative pressure above the glucose solution from pulling a ventilation syringe to 10 cm^3^ as shown in Fig. 1C (see further explanation in ref. [22]). The corresponding inhaled volume of 8 cm^3^ was measured through exhalation causing water displacement in a water bell.

### 2.4 MRI protocol

MRI experiments were performed using a vertical bore 9.4 T Bruker Avance III microimaging system (Bruker Corporation, Billerica, Massachusetts, USA). Imaging experiments utilized a Bruker 30 mm double saddle probe tuned to 15.4 MHz corresponding to the resonance frequency of ^83^Kr gas in the lung. Images were acquired by means of N = 32 phase encoding gradient increments using a variable flip angle (VFA) FLASH protocol (TE = 4.2 ms, TR = 19.2 ms) that reduced the effects of T_1_ decay; the flip angle of the i^th^ increment (*θ*_i_) was calculated by $\theta_{i}\approx \tan^{-1}\left(1/\sqrt{N-i}\right)$ [23]. The imaging protocol had a total acquisition time 0.615 s limiting the T_1_ decay during acquisition.

**Fig. 3:**
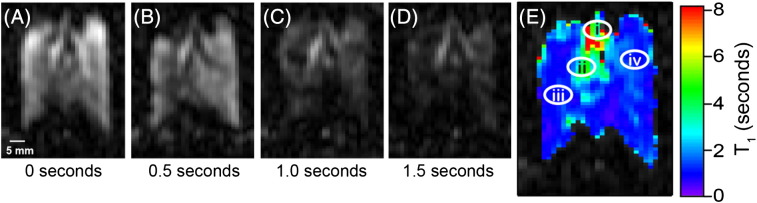
Series of hp ^83^Kr MR images demonstrating SQUARE contrast. A new delivery of hp ^83^Kr was provided for each image shown. (A) VFA FLASH MRI as in Fig. 2b but with 3 mm slice selection. (B–D) MR images as in (A) with a relaxation delay, t_d_, between hp gas inhalation and acquisition as indicated in the figure. The major airways are visibly less affected than the alveolar space by increasing t_d_ values. (E) Graphical representation of the T_1_ values calculated from the signal decay in (A – D) for each volume element (voxel). Decay curves for each of the voxels located at positions i–iv in (E) are shown in Fig. 4.

Coronal images were acquired into 64 × 32 matrices resulting in a field of view (FOV) of 50.9 mm in the longitudinal (frequency encoding) and 40.7 mm in the transverse (phase encoding) directions, respectively. To acquire a non-slice selective image, 0.3 ms rectangular hard pulses of variable power levels were used for excitation. The slice selective images utilize 2 ms sinc-shaped radio frequency pulses of variable power to selectively excite a 3 mm central coronal slice of the lung, resulting in a nominal resolution of 0.80 × 1.27 × 3 mm^3^. To obtain T_1_-weighted images and demonstrate SQUARE pulmonary MRI contrast the imaging sequence was started with a programmed time delay (t_d_) of 0.0 s, 0.5 s, 1.0 s or 1.5 s after inhalation. The inhalation itself was accomplished manually by reducing the pressure in the artificial pleural cavity using the ventilation syringe as described in ref. [22]. Slight alternations in the timing (approximately ± 0.2 s) of the manual inhalation procedure were deemed acceptable. Note that the uncertainty in the exact timing of the images can be eliminated by future improved MRI protocols that record multiple images within one inhalation cycle. In this work, each individual image was acquired from a single inhalation cycle and subsequent VFA FLASH acquisition (NEX = 1) with no signal averaging. Slice selective images demonstrating SQUARE MRI contrast (Fig. 3A–D) and the resulting T_1_ map (Fig. 3E) were acquired using a single animal.

### 2.5 Image reconstruction and analysis

Images were processed and reconstructed in Prospa (v. 3.06, Magritek, Wellington, New Zealand) by applying a sine-bell squared window function to the raw data before two-dimensional Fourier transformation. The two dimensional image data were exported for further analysis using IGOR Pro (v. 6.01; Wavemetrics, Lake Oswego, OR, USA).

**Fig. 4:**
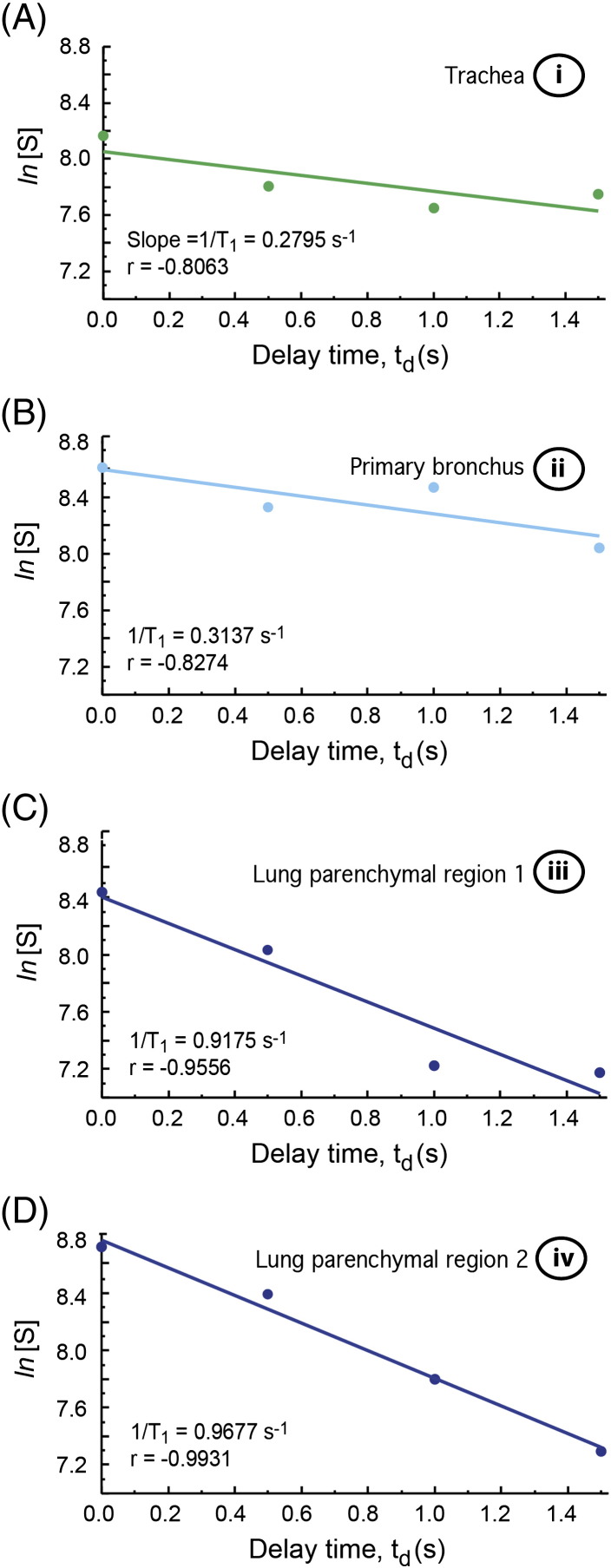
Semi-logarithmic plots of signal decay as a function of delay time, t_d_, in the excised lung. Data were selected from various anatomical locations as indicated in Fig. 3E: (A) tracheal region (i); (B) major bronchial region (ii); (C) and (D) lung parenchymal regions (iii) and (iv), respectively. Colors are in accordance with Fig. 3E. Linear correlation coefficients, or Pearson's r, and relaxation rates are shown in the annotation for each plot.

To construct the T_1_ map shown in Fig. 3E the image data were combined into a three dimensional matrix having two spatial dimensions (the slice selective images) and one time dimension (the delay before acquisition). Linear regression analysis of the natural logarithm of the signal intensity as a function of delay time was used to obtain spatially resolved T_1_ values in Fig. 3E. Representative data from four selected volume elements in Fig. 3E are shown in Fig. 4. T_1_ values calculated outside the lung region were composed solely of background noise and were not displayed in Fig. 3E. The final T_1_ map was overlaid onto the lung image at delay time t_d_ = 0 s for clarity of presentation.

### 2.6 Animal care and preparation

Male Sprague–Dawley rats (350–400 g, Charles River UK Ltd, Margate, UK) were euthanized by overdose of pentobarbital (Sigma-Aldrich Ltd, Gillingham, UK) in accordance with local animal welfare guidelines and the Animals (Scientific Procedures) Act (1986). Immediately after confirmation of death, a catheter was inserted into the caudal vena cava to allow flushing of the pulmonary circulation with 20–30 cm^3^ heparin 100 IU/cm^3^ (Wockhardt UK Ltd, Wrexham, UK) in 0.9% saline solution (Baxter Healthcare Ltd, Thetford, UK) followed with phosphate buffer solution (PBS, Sigma-Aldrich Ltd, Gillingham, UK) in order to remove residual blood from the pulmonary circulation.

The heart and lungs were removed *en masse*. A polytetrafluorethylene (PTFE) adapter tube was inserted 5–10 mm above the carina and sutured into place. The heart and lungs were suspended in 5% glucose solution (weight/volume) with the trachea pointing downwards in a custom-built acrylic ventilation chamber, as detailed in Fig. 1. The ex vivo lungs were repeatedly inflated with 8–10 cm^3^ of room air to check for leakage either from the suture around the trachea or the lungs themselves. For the presented work the lung harvesting procedure was completed with 100% success of removing the lungs intact. Normally with a skilled operator the ex vivo technique results in over 90% of lungs being suitable for imaging. The lungs were chilled to 278 K for transportation to the imaging facility.

## 3 Results and discussion

The pure gas phase relaxation time of ^83^Kr is sufficiently long with T_1_ times of several minutes at ambient pressure [16] to permit hp gas extraction and transfer. However, as the ^83^Kr relaxation is accelerated by the presence of surfaces, the contact of the hp gas with any material during this process needs to be minimized. Pumps that have been used for extraction and compression of ^3^He after metastable exchange optical pumping (MEOP) [24] typically require many compression cycles to transfer the entire hp gas volume [24–27]. For the extraction and compression of the quadrupolar hp ^83^Kr a pneumatically operated piston within a large volume cylinder was designed that used a single extraction–compression cycle as shown in Fig. 1.

This design is conceptually similar to the gas pressure driven ‘syringe’ using a Teflon piston as applied previously by Rosen et al. [28] for the transfer of hp ^129^Xe following cryogenic gas separation. However, the extraction unit in this work needed to attain vacuum conditions of less than 0.2 kPa prior to hp gas extraction from the SEOP cell and, following extraction, was required to compress the hp gas to ambient pressure. Therefore, this unit operates at a high pressure differential and an O-ring seal equipped acrylic piston provides gas tight isolation of the two compartments of the extraction unit. The setup allowed for the extraction of about 3/4 of the hp gas from the SEOP cell in a single expansion–compression cycle. The losses in polarization caused by compression, shown in Fig. 2A, were negligible at SEOP pressures above 75 kPa and were still acceptable down to 50 kPa. Using a 25% krypton–75% N_2_ mixture for a SEOP duration of 8 minutes at a pressure of 50 kPa, the apparent spin polarization *P*_app_ = 2.9% was found after extraction and transfer of the hp gas into a sample cell as seen in Fig. 2.

For the MRI, an SEOP cell pressure of 90–100 kPa was used, even though the attained apparent polarization of *P*_app_ = 2.0% was only about 2/3 the maximum possible value (Fig 2A, red arrow). The higher SEOP pressure ensured that the quantity of the produced hp gas (i.e. 40 cm^3^ hp gas at ambient pressure) was sufficient to match the actual inhaled volume and the dead volume in the gas transfer system.

After SEOP with isotopically enriched ^83^Kr followed by extraction, compression, and delivery of the gas mixture into the (ambient pressure) storage chamber (V_B_) located underneath the breathing apparatus, 8 cm^3^ of the hp gas was inhaled by the excised lungs using the breathing apparatus shown in Fig. 1B and C (see also ref. [22]). The signal intensity was sufficient to provide anatomical details, such as the shape of the lung lobes and the distinction of major airways, using a variable flip angle (VFA) FLASH MRI protocol [23] without slice selection but also without signal averaging having SNR = 51 as shown in Fig. 2B. Further experimental details of the MRI protocol, animal usage and SEOP are described in the Materials and methods section.

After the addition of 3 mm slice selection to the VFA FLASH MRI protocol, the major airways could clearly be recognized in a single acquisition (i.e. NEX = 1) as show in Fig. 3A. Furthermore, the obtained signal intensity was sufficient to permit the proof of principle study of ^83^Kr SQUARE contrast in lungs. Fig. 3B–D shows the same 3 mm slice selective hp ^83^Kr images as Fig. 3A, but with a delay period t_d_ between inhalation and start of the image acquisition ranging from 0.5 s to 1.5 s (t_d_ = 0 s in Fig. 3A). A new bolus of hp ^83^Kr was delivered for each of the images. As a clear trend observed directly in these four images (Fig. 3A–D), the signal originating from the major airways was less affected by the delay time than the rest of the lung. The cause for the slower relaxation was presumably the smaller surface to volume (S/V) ratio in the airways as opposed to the alveolar space.

Smaller airways were not resolved but contribute to the contrast observed in the MR images. Fig. 3E shows a T_1_ relaxation time map obtained from the t_d_ dependent signal decay of each volume element in Fig. 3A–D. The longitudinal relaxation time (averaged over 20 voxel) for the trachea is T_1_ = 5.3 ± 1.9 s and T_1_ = 3.0 ± 0.9 s for the main stem bronchus. The averaged relaxation times measured in lung parenchyma adjacent to the major airways and in the periphery of the lung are T_1_ = 1.1 ± 0.2 s and T_1_ = 0.9 ± 0.1 s respectively. The signal decays of selected voxel are shown in Fig. 4. The observed T_1_ data are in reasonable agreement with previous, spatially unresolved bulk measurements of ^83^Kr T_1_ relaxation in excised rat lungs that also demonstrated that the addition of up to 40% of O_2_ did not significantly alter the T_1_ times [22].

SQUARE originates from surfaces but its effect is detected in the gas phase due to rapid exchange. It is however not known to what depth the alveolar surface, which is comprised of surfactant molecules and proteins, followed by a water layer, cell tissue, and the vascular system (filled with phosphate buffer solution in this work), is probed by the SQUARE effect. The relaxation of the krypton dissolved in extracellular water is too slow, i.e. T_1_ = 100 ms at 298 K [29], to be a major contributor to the observed T_1_ values in the alveolar region, given the small quantity of krypton dissolved in extracellular water. SQUARE may therefore originate from a deeper layer (i.e. cell tissue) or may be caused by interactions of the krypton atoms with the outer surfactant layer. The answer to this question could have profound impact on potential usage of SQUARE for disease related contrast but its exploration is beyond the scope of this work.

As Figs. 2 and 3 demonstrate, the extraction technique from low pressure (90–100 kPa) SEOP cells works well, generating reproducibly *P*_app_ = 2.0% with a line narrowed laser providing 23.3 W of power incident at the SEOP cell. This resulted in an approximately 10 fold increase in MR signal intensity as compared to the previously published results on hp ^83^Kr MRI in excised rat lungs [19]. An additional factor of 8.7 improvement in signal to noise ratio was achieved by using isotopically enriched to 99.925% ^83^Kr gas. Not surprisingly for a spin system with *P*_app_ = 2%, the obtained resolution fell short compared to ventilation hp ^129^Xe MRI. However, the ^83^Kr signal intensity was strong enough to allow for surface sensitive contrast in excised lungs while retaining structural resolution. The voxel resolution obtained with the slice selective hp ^83^Kr MRI is 0.80 × 1.27 × 3 mm^3^, (SNR = 23.8 for t_d_ = 0 s) and is therefore similar to dissolved phase ^129^Xe pulmonary MRI that uses the small fraction (typically 1–2%) of inhaled xenon dissolved in tissue and blood.

The applied laser power of 23.3 W (incident at the SEOP cell) can be increased significantly due to recent advances in solid state laser technology and may thus improve the quantity of the produced hp gas and its spin polarization. Larger volume SEOP cells could be used to produce larger quantities of hp gas volumes at lower pressures if the power density of the laser irradiation is maintained across the larger cross section. Alternatively, the volume of hp gas can also be increased if several SEOP units of the current cell size and laser power operate in parallel. The amount of hp gas needed per inhalation cycle may additionally be reduced by optimizing the ambient pressure storage container (V_B_), consequently allowing for lower SEOP cell pressures that result in higher spin polarization with the current setup.

A potential drawback of the presented methodology is that the lungs may become contaminated by rubidium vapors during the rapid delivery of hp gas from the SEOP cell. Therefore, the extraction unit was tested at various locations for rubidium residues through pH measurements (ColorpHast). Although more elaborate testing is required, and it appears that most of the rubidium tends to condense in the tubing located before the extraction unit. The use of additional rubidium filters that make use of the high reactivity of the alkali metal may improve the situation further but was not explored.

## 4 Conclusions

Using improved hp ^83^Kr production methodology, SQUARE MRI contrast was demonstrated between airways and alveolar regions. Lung pathology related contrast was not attempted as animal models of pulmonary disease were beyond the scope of this proof of concept study. However, the produced signal intensity will be sufficient to attempt disease specific contrast in pathophysiology and to explore whether hp ^83^Kr is of supplemental diagnostic value to hp ^3^He and hp ^129^Xe MRI. The potential usage of hp ^83^Kr as a novel contrast agent should be investigated for disorders such as emphysema where the lung surface to volume ratio (S/V) is reduced [30,31], or generally for the broad spectrum of diseases which exhibit significant changes in lung surface chemistry, for example acute lung injury (ALI), acute respiratory syndrome (ARDS) [32] and cystic fibrosis (CF) [33]. Two final notes with regard to practicalities of hp ^83^Kr MRI: (1) Krypton gas (natural abundance of 11.5% ^83^Kr) is a renewable resource generated as a by-product of air liquefaction, available at approximately €1 per liter (at ambient pressure). Unfortunately, isotopically enriched ^83^Kr is costly (approximately € 4000/L) at the current low demand for production. (2) There are little toxicological concerns for future clinical applications as krypton is chemically inert and does not exhibit anesthetic properties at ambient gas pressure [34,35].
